# Mapping EORTC QLQ-C30 and FACT-G onto EQ-5D-5L index for patients with cancer

**DOI:** 10.1186/s12955-020-01611-w

**Published:** 2020-11-03

**Authors:** Yasuhiro Hagiwara, Takeru Shiroiwa, Naruto Taira, Takuya Kawahara, Keiko Konomura, Shinichi Noto, Takashi Fukuda, Kojiro Shimozuma

**Affiliations:** 1 Department of Biostatistics, Division of Health Sciences and Nursing, The University of Tokyo, 7-3-1, Hongo, Bunkyo-ku, Tokyo, 113-0033 Japan; 2 Center for Outcomes Research and Economic Evaluation for Health, National Institute of Public Health, Wako, Japan; 3 Breast and Endocrine Surgery Department, Okayama University Hospital, Okayama, Japan; 4 Clinical Research Promotion Center, The University of Tokyo Hospital, Tokyo, Japan; 5 Center for Health Economics and QOL Research, Niigata University of Health and Welfare, Niigata, Japan; 6 Department of Biomedical Sciences, College of Life Sciences, Ritsumeikan University, Kusatsu, Japan

**Keywords:** Cancer, EORTC QLQ-C30, EQ-5D-5L, FACT-G, Mapping, Preference-based measure

## Abstract

**Background:**

To develop direct and indirect (response) mapping algorithms from the European Organization for Research and Treatment of Cancer Quality of Life Questionnaire Core 30 (EORTC QLQ-C30) and the Functional Assessment of Cancer Therapy General (FACT-G) onto the EQ-5D-5L index.

**Methods:**

We conducted the QOL-MAC study where EQ-5D-5L, EORTC QLQ-C30, and FACT-G were cross-sectionally evaluated in patients receiving drug treatment for solid tumors in Japan. We developed direct and indirect mapping algorithms using 7 regression methods. Direct mapping was based on the Japanese value set. We evaluated the predictive performances based on root mean squared error (RMSE), mean absolute error, and correlation between the observed and predicted EQ-5D-5L indexes.

**Results:**

Based on data from 903 and 908 patients for EORTC QLQ-C30 and FACT-G, respectively, we recommend two-part beta regression for direct mapping and ordinal logistic regression for indirect mapping for both EORTC QLQ-C30 and FACT-G. Cross-validated RMSE were 0.101 in the two methods for EORTC QLQ-C30, whereas they were 0.121 in two-part beta regression and 0.120 in ordinal logistic regression for FACT-G. The mean EQ-5D-5L index and cumulative distribution function simulated from the recommended mapping algorithms generally matched with the observed ones except for very good health (both source measures) and poor health (only FACT-G).

**Conclusions:**

The developed mapping algorithms can be used to generate the EQ-5D-5L index from EORTC QLQ-C30 or FACT-G in cost-effectiveness analyses, whose predictive performance would be similar to or better than those of previous algorithms.

## Background

Cancer is a common disease in many countries in the twenty-first century; there were estimated 18.1 million new cancer cases and 9.6 million cancer deaths in 2018 [1]. Although the advancements in cancer treatment prolong life and improve the quality of life of patients, the fight against cancer seems to have a long way to go. One recent problem related to cancer treatments is their cost. In the era of targeted, immune, and gene therapies, some treatments are highly effective but costly [2]. With limited medical resources, we need to evaluate not only the effectiveness of cancer treatments but also their cost-effectiveness [3].

In cost-effectiveness analyses of cancer treatments, the most important and commonly used health outcome is quality-adjusted life year (QALY). QALY incorporates both the duration and quality of life, the two most important aspects for patients with cancer, and enables us to compare the cost-effectiveness of treatments in resource allocation irrespective of the disease area [4]. Calculation of QALY requires health utility data that are used as weights for quality of life. Since direct elicitation of health utility is burdensome, multi-attribute preference-based measures, such as EQ-5D are often used [5]. The National Institute for Health and Care Excellence in the United Kingdom prefers the use of EQ-5D in cost-effectiveness analyses [6], whereas the Center for Outcomes Research and Economic Evaluation for Health in Japan recommends a preference-based measure based on the time trade-off method [7, 8], which virtually indicates EQ-5D in the current situation. Since EQ-5D with 3 levels (EQ-5D-3L) has several limitations, such as ceiling effect and multimodality [9, 10], EQ-5D with 5 levels (EQ-5D-5L) was developed and the value sets for it are now available in many countries [11].

Unfortunately, the EQ-5D data is often directly unavailable when cost-effectiveness analyses for cancer treatments are conducted. Instead, cancer-specific non-preference-based health-related quality of life (HRQOL) measures that are directly relevant and sensitive to cancer-related treatments and symptoms are used to evaluate the HRQOL of patients with cancer. The two common cancer-specific HRQOL measures are the European Organization for Research and Treatment of Cancer Quality of Life Questionnaire Core 30 (EORTC QLQ-C30) and the Functional Assessment of Cancer Therapy General (FACT-G) [12, 13]. Although many mapping algorithms from the two cancer-specific measures onto the EQ-5D-3L index have been developed [14–16], there are just a few onto EQ-5D-5L [17–21]. Based on our literature survey and the latest mapping algorithm database [22], we find that there are no direct mapping algorithms for EQ-5D-5L based on the Japanese value set and no indirect (response) mapping algorithms for EQ-5D-5L from EORTC QLQ-C30 and FACT-G.

The aim of the present study is to develop direct and indirect mapping algorithms from EORTC QLQ-C30 and FACT-G onto the EQ-5D-5L index using data from the Quality Of Life Mapping Algorithm for Cancer (QOL-MAC) study, where EQ-5D-5L, EORTC QLQ-C30, and FACT-G data are cross-sectionally obtained from patients with cancer.

## Methods

### Study design and patients

We conducted the QOL-MAC study, a multicenter, cross-sectional study to develop mapping algorithms for EORTC QLQ-C30 and FACT-G onto the EQ-5D-5L index. This study was conducted in 14 hospitals (all participating hospitals are listed in Additional file 1) in Japan from November 2018 to March 2019. The target sample size (1200 patients) was not formally based on statistical considerations. This study was conducted in accordance with the Declaration of Helsinki and the study protocol was approved by each participating hospital.

We enrolled patients with locally advanced, metastatic, or recurrent cancer with the following eligibility criteria: aged 20 or above; with lung, stomach, colorectal, or breast cancer, or any other solid tumor; under drug therapy; and with an Eastern Cooperative Oncology Group (ECOG) performance status of 0–3. We excluded those who received treatment for multiple primary tumors or who are not able to respond to the questionnaires. We recruited both outpatients and inpatients to collect a variety of data on health status that patients with cancer could experience. All enrolled patients gave written informed consent before study enrollment.

### Instruments

We conducted the EQ-5D-5L assessment using the Japanese version of the EQ-5D-5L questionnaire [11, 23], which has 5 items: mobility, self-care, usual activities, pain/discomfort, and anxiety/depression. In EQ-5D-5L, patients are asked to assign a status level (from the 5 given) to each item: no problem, slight problem, moderate problem, severe problem, or extreme problem (the wording is slightly different among the five items). We converted the responses to EQ-5D-5L into the EQ-5D-5L index using the Japanese value set based on the time trade-off method [23]. The indexes of 0 and 1 represent death and full health, respectively.

We assessed HRQOL using the Japanese versions of the EORTC QLQ-C30 questionnaire (version 3) and the FACT-G questionnaire (version 4) as the source measures in mapping algorithms [12, 13]. EORTC QLQ-C30 has 30 items and the responses to those items were converted into 5 functioning subscale scores, 9 symptom subscale scores, and a global health status score. Higher scores on the functioning subscales and global health status indicate better health condition, whereas higher scores on symptom subscales indicate severer symptom. FACT-G has 27 items and the responses to those were converted into 4 well-being subscale scores. Higher scores on the 4 well-being subscales indicate better health condition.

We asked the participants to complete a combined questionnaire of the three instruments. In addition to the three instruments, we collected data on patients’ demographic and clinical characteristics.

### Statistical analysis

We defined the analysis population for EORTC QLQ-C30 as eligible patients having both EQ-5D-5L index and all 15 subscale scores and for FACT-G as eligible patients having both EQ-5D-5L index and all 4 subscale scores. We summarized the patient characteristics and responses to EQ-5D-5L, EORTC QLQ-C30, and FACT-G in each analysis population. As a preliminary assessment of the conceptual overlap of the two source measures to EQ-5D-5L, we calculated Spearman’s rank correlation coefficients between the subscale scores of the two source measures and the responses to the five items in EQ-5D-5L.

We developed the mapping algorithms for each source measure using 7 regression methods. Based on qualitative and quantitative assessments of the conceptual overlap between the source and target measures, all 5 functioning subscales, global health status, and two symptom subscales (fatigue and pain) were selected as initial candidate variables for direct mapping of EORTC QLQ-C30, and all 4 well-being subscales were selected as initial candidate variables for direct mapping of FACT-G. For indirect mapping, we selected subscales that had an absolute rank correlation of ≥ 0.4 (EORTC QLQ-C30) and ≥ 0.3 (FACT-G) for each EQ-5D-5L item as initial candidate variables. Furthermore, we included age and sex into the initial candidate variables in all regression methods. We selected explanatory variables using the backward selection method, which sequentially omitted variables with the largest *P* value > 0.15. This *P* value criterion approximately corresponds to the backward selection based on the Akaike information criterion [24]. No higher-order terms or interaction terms were considered.

The seven regression methods were linear regression, beta regression [25], tweedie regression [26], tobit regression, two-part linear regression, two-part beta regression [27], and ordinal logistic regression. All the regression methods except ordinal logistic regression were directly applied to the EQ-5D-5L index, whereas ordinal logistic regression was applied to each EQ-5D-5L item and used to develop the indirect mapping algorithms. In beta regression, we transformed the EQ-5D-5L index to {observed index − (− 0.025)}/{1 − (− 0.025)} (− 0.025 is the lowest index in the Japanese value set) [25]. In tweedie regression, we transformed the EQ-5D-5L index into disutility from full health (i.e., 1 − observed index). In tobit regression, we set the lower and upper bounds of − 0.025 and 1, respectively. In two-part regression methods, we predicted full health using logistic regression. In two-part beta regression, we transformed the EQ-5D-5L index to {observed index − (− 0.025)}/{0.895 − (− 0.025)} (0.895 is the second largest index in the Japanese value set). In beta and two-part beta regressions, we added 0.005 and subtracted 0.005 at the lower and upper bounds, respectively [28]. We calculated the predicted EQ-5D-5L index as an expected value provided by the fitted models. For ordinal logistic regression, the predicted EQ-5D-5L index was calculated as 1 minus the sum of disutilities of the 5 levels weighted by the predicted probabilities over the 5 items.

We first evaluated the performance of our mapping algorithms based on root mean squared error (RMSE), mean absolute error (MAE), and Pearson’s correlation coefficient between the observed and predicted EQ-5D-5L indexes. These measures were calculated for the whole sample and ninefold cross validation. In the cross-validation, we randomly divided the whole sample into 9 subsamples (approximately 100 patients in each subsample); repeatedly conducted variable selection in 8 subsamples and calculated the performance measures for the remaining subsample; and averaged them in subsamples to compute overfitting-corrected performance measures.

After selecting the mapping algorithms with a good predictive performance in terms of the above three measures, we checked the selected mapping algorithms in terms of face validity. We eliminated any explanatory variables that had regression coefficients with a sign that was the opposite of what was anticipated and *P* > 0.05, and re-estimated the regression models to obtain the final mapping algorithms. We simulated the EQ-5D-5L index from the selected final mapping algorithms and compared the mean observed and simulated EQ-5D-5L indexes in various subgroups. Furthermore, we plotted the cumulative distribution functions of observed and simulated EQ-5D-5L indexes.

All statistical analyses were conducted using Base SAS, SAS/STAT, or SAS/ETS software, Version 9.4 of the SAS System for Windows.

## Results

### Patients and descriptive data

A total of 1031 patients were enrolled into the QOL-MAC study. Of the 1029 eligible patients, 903 and 908 patients were included in the EORTC QLQ-C30 analysis population and FACT-G analysis population, respectively. Table 1 shows patients’ characteristics in the two analysis populations. Lung cancer and colorectal cancer were two major tumor types; 21% of the patients were hospitalized; and 65% were receiving chemotherapy.

**Table 1:** Patient characteristics in the two analysis populations

Characteristic	EORTC QLQ-C30	FACT-G
	N = 903	N = 908
Age (years)	68 (58–74)	68 (58–74)
Sex		
Male	489 (54.2)	489 (53.9)
Female	414 (45.8)	419 (46.1)
Hospitalization		
Yes	186 (20.6)	180 (20.8)
No	717 (79.4)	719 (79.2)
ECOG performance status		
0	451 (49.9)	447 (49.2)
1	368 (40.8)	377 (41.5)
2	63 (7.0)	64 (7.0)
3	20 (2.2)	19 (2.1)
Unknown (0, 1, 2, or 3)	1 (0.1)	1 (0.1)
Tumor type		
Lung cancer	317 (35.1)	312 (34.4)
Stomach cancer	65 (7.2)	64 (7.0)
Colorectal cancer	222 (24.6)	226 (24.9)
Breast cancer	113 (12.5)	116 (12.8)
Other solid tumors	186 (20.6)	190 (20.9)
Stage at diagnosis		
I	49 (5.4)	48 (5.3)
II	70 (7.8)	70 (7.7)
III	184 (20.4)	185 (20.4)
IV	588 (65.1)	593 (65.3)
Unknown	12 (1.3)	12 (1.3)
Site of metastasis or recurrence^a^		
None	77 (8.5)	81 (8.9)
Liver	198 (21.9)	204 (22.5)
Lung	285 (31.6)	280 (30.8)
Bone	160 (17.7)	165 (18.2)
Brain	98 (10.9)	98 (10.8)
Lymph nodes	361 (40.0)	361 (39.8)
Others	225 (24.9)	227 (25.0)
History of surgery		
Yes	480 (53.2)	483 (53.2)
No	422 (46.7)	424 (46.7)
Unknown	1 (0.1)	1 (0.1)
Type of treatment^a^		
Chemotherapy	582 (64.5)	590 (65.0)
Endocrine therapy	65 (7.2)	66 (7.3)
Molecular targeted therapy	159 (17.6)	158 (17.4)
Immunotherapy	108 (12.0)	104 (11.5)
Palliative therapy	35 (3.9)	37 (4.1)
Others	8 (0.9)	8 (0.9)

Median (IQR) is reported for age, whereas number (%) is reported for other characteristics

EORTC QLQ-C30, European Organization for Research and Treatment of Cancer Quality of Life Questionnaire Core 30; FACT-G, Functional Assessment of Cancer Therapy General; ECOG, Eastern Cooperative Oncology Group

^a^Multiple choices are allowed

Table 2 shows the distributions of the EQ-5D-5L index and subscale scores of EORTC QLQ-C30 and FACT-G. The mean EQ-5D-5L index was 0.781 in both analysis populations. We provided the data on the response to each EQ-5D-5L item in Additional file 1: Table S3. The correlations between the subscale scores of the two source measures and the responses to the five items in EQ-5D-5L are reported in Additional file 1: Table S4. All functioning subscales except cognitive functioning, global health status, fatigue, pain, and appetite loss in EORTC QLQ-C30 had an absolute rank correlation coefficient of ≥ 0.4 in at least one item, whereas physical well-being, emotional well-being, and functional well-being subscales in FACT-G had an absolute rank correlation coefficient of ≥ 0.3 in at least one item.

**Table 2:** Distributions of the EQ-5D-5L index and subscale scores of EORTC QLQ-C30 and FACT-G

	Minimum	5%	25%	Median	75%	95%	Maximum
EORTC QLQ-C30 analysis							
EQ-5D-5L index (Japan)	− 0.025	0.437	0.691	0.823	0.895	1	1
EORTC QLQ-C30							
Physical functioning	0	33.3	66.7	86.7	93.3	100	100
Role functioning	0	0	66.7	83.3	100	100	100
Emotional functioning	0	44.4	75	83.3	100	100	100
Cognitive functioning	0	33.3	66.7	83.3	100	100	100
Social functioning	0	33.3	66.7	83.3	100	100	100
Global health status	0	16.7	50	66.7	83.3	100	100
Fatigue	0	0	22.2	33.3	55.6	77.8	100
Nausea and vomiting	0	0	0	0	16.7	33.3	100
Pain	0	0	0	16.7	33.3	66.7	100
Dyspnea	0	0	0	33.3	33.3	100	100
Insomnia	0	0	0	33.3	33.3	66.7	100
Appetite loss	0	0	0	0	33.3	100	100
Constipation	0	0	0	33.3	33.3	66.7	100
Diarrhea	0	0	0	0	33.3	66.7	100
Financial difficulties	0	0	0	33.3	33.3	100	100
FACT-G analysis							
EQ-5D-5L index (Japan)	− 0.025	0.436	0.691	0.823	0.895	1	1
FACT-G							
Physical well-being	1	9	16	22	25	28	28
Social/family well-being	0	5	14	18	22	26.8	28
Emotional well-being	0	7	13	17	20	23	24
Functional well-being	0	6	13	17	22	27	28

EORTC QLQ-C30, European Organization for Research and Treatment of Cancer Quality of Life Questionnaire Core 30; FACT-G, Functional Assessment of Cancer Therapy General

### Model selection and selected models

Table 3 shows the predictive performance of the fitted models. For EORTC QLQ-C30, two-part beta regression provided the best model in all three measures for the whole sample, whereas linear regression provided the best model in all three measures for cross-validation. The difference in the predictive performance was marginal between linear regression and two-part beta regression in the whole sample and cross-validation. Ordinal logistic regression had a performance that was comparable to these models for the whole sample and cross-validation. For FACT-G, two-part beta regression and ordinal logistic regression provided the best model in all three measures for the whole sample, whereas ordinal logistic regression provided the best model in all three measures for cross-validation. After inspecting face validity, the emotional well-being subscale was eliminated from the ordinal logistic regression for usual activities in FACT-G analysis. The three performance measures in whole sample after elimination of the emotional well-being were the same in the display digits as in Table 3.

**Table 3:** Predictive performance of each mapping algorithm

	Whole sample			ninefold cross-validation		
	RMSE	MAE	*ρ*	RMSE	MAE	*ρ*
EORTC QLQ-C30						
Linear	*0.099*	*0.075*	0.838	*0.100*	*0.076*	*0.833*
Beta	0.103	0.081	0.825	0.105	0.081	0.817
Tweedie	0.110	0.084	0.803	0.114	0.086	0.799
Tobit	0.102	0.077	0.836	0.103	0.078	0.822
Two-part linear	0.100	*0.075*	0.837	0.101	*0.076*	0.825
Two-part beta	*0.099*	*0.075*	*0.840*	0.101	0.077	0.828
Ordinal logistic	0.100	0.077	0.835	0.101	0.078	0.829
FACT-G						
Linear	0.121	*0.090*	0.753	0.121	*0.091*	0.744
Beta	0.121	0.091	0.754	0.122	0.092	0.752
Tweedie	0.124	0.092	0.740	0.124	0.093	0.740
Tobit	0.123	*0.090*	0.754	0.124	*0.091*	0.751
Two-part linear	0.122	0.091	0.749	0.123	0.092	0.748
Two-part beta	*0.119*	*0.090*	*0.760*	0.121	*0.091*	0.759
Ordinal logistic	*0.119*	*0.090*	*0.760*	*0.120*	*0.091*	*0.764*

The best performances in each performance measure in each source measure are italics

RMSE, root mean squared error; MAE, mean absolute error; *ρ*, correlation coefficient; EORTC QLQ-C30, European Organization for Research and Treatment of Cancer Quality of Life Questionnaire Core 30; FACT-G, Functional Assessment of Cancer Therapy General

**Fig. 1:**
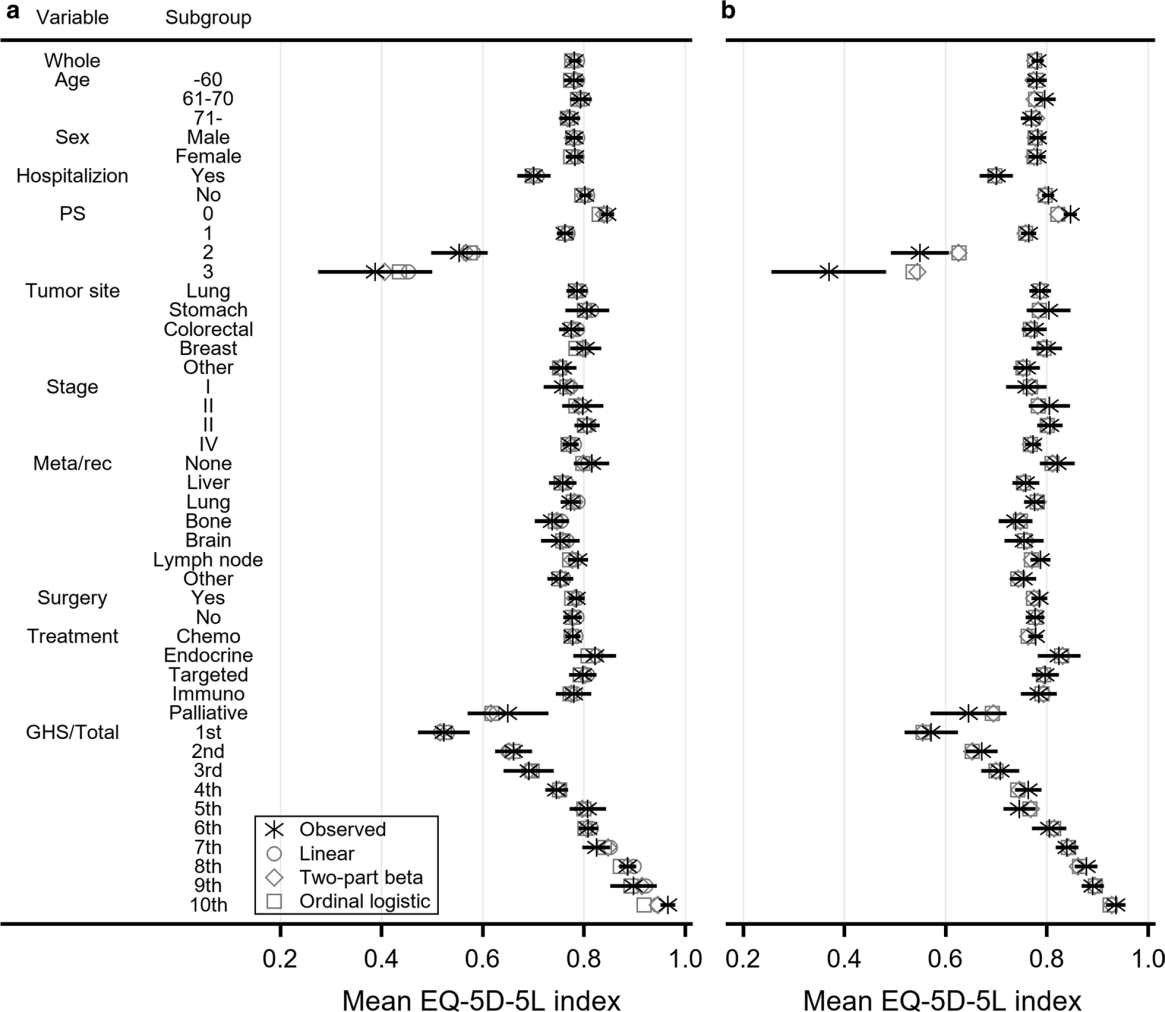
Observed and simulated mean EQ-5D-5L index in various subgroups for **a** EORTC QLQ-C30, **b** FACT-G. Error bar represents 95% confidence interval of observed mean EQ-5D-5L index. Global health status and FACT-G total scores were used to define 10 subgroups for EORTC QLQ-C30 and FACT-G, respectively. EORTC QLQ-C30, European Organization for Research and Treatment of Cancer Quality of Life Questionnaire Core 30; FACT-G, Functional Assessment of Cancer Therapy General; Meta/rec, Metastasis/recurrence; GHS/Total, global health status/FACT-G total

**Fig. 2:**
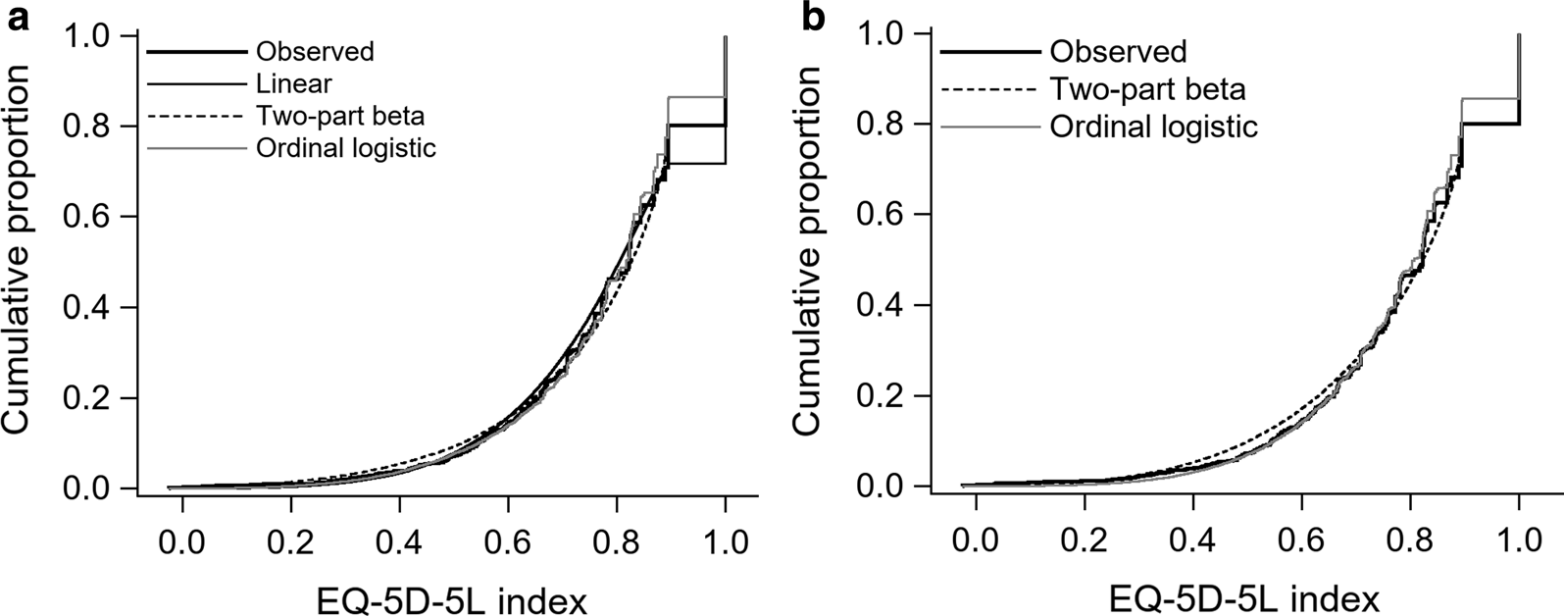
Cumulative distribution functions of observed and simulated EQ-5D-5L index for **a** EORTC QLQ-C30, **b** FACT-G. EORTC QLQ-C30, European Organization for Research and Treatment of Cancer Quality of Life Questionnaire Core 30; FACT-G, Functional Assessment of Cancer Therapy General

Figure 1 depicts the mean EQ-5D-5L index simulated from the best-performed mapping algorithms against the observed mean EQ-5D-5L in various subgroups. The three mapping algorithms (linear, two-part beta, and ordinal logistic regression) for EORTC QLQ-C30 were well calibrated except for the subgroup with the highest global health status score, whereas the two mapping algorithms (two-part beta and ordinal logistic regression) for FACT-G provided an overestimated mean EQ-5D-5L index in the subgroups with an ECOG performance status of 2 and 3. Figure 2 shows the cumulative distribution functions of the observed and simulated EQ-5D-5L. The mapping algorithms based on two-part beta regression predicted more EQ-5D-5L index below 0.6 and less EQ-5D-5L index between 0.6 to 0.9 than the observed EQ-5D-5L data. The mapping algorithms based on ordinal logistic regression provided a smaller proportion of full health than the observed EQ-5D-5L data. These features were applicable to both EORTC QLQ-C30 and FACT-G. For EORTC QLQ-C30, the mapping algorithm based on linear regression provided a larger proportion of full health than the true EQ-5D-5L data.

Based on the above evaluations, we recommend two-part beta regression for direct mapping algorithms and ordinal logistic regression for indirect mapping algorithms for both EORTC QLQ-C30 and FACT-G. Liner regression for EORTC QLQ-C30 is not recommended due to overestimation of the proportion of full health described in Fig. 2. Table 4 shows the estimated regression coefficients. Uncertainty in the estimated regression coefficients is provided in Additional file 1: Tables S5–S8 in the form of variance covariance matrix. We also provided the detailed calculation of the EQ-5D-5L index from these mapping algorithms in the Additional file 1.

**Table 4:** Regression coefficients for the recommended mapping algorithms

	Two-part beta		Ordinal logistic				
	Logistic part	Beta part	Mobility	Self-care	Usual activities	Pain/discomfort	Anxiety/depression
EORTC QLQ-C30							
Intercept 1	− 11.64060	− 1.66963	− 5.91277	− 4.24535	− 9.28712	− 0.82400	− 6.65962
Intercept 2	–	–	− 3.79148	− 2.56552	− 6.21474	2.75434	− 4.31221
Intercept 3	–	–	− 1.85222	− 1.23110	− 4.16801	5.35925	− 2.81811
Intercept 4	–	–	0.92106	0.17985	− 1.46187	7.64850	− 0.86976
Age	–	–	− 0.02469^a^	–	–	–	0.02119^a^
Female	–	0.15271	–	0.60145^a^	0.33291	–	–
Physical functioning	0.03395^a^	0.02475^a^	0.06934^a^	0.06035^a^	0.04812^a^	–	–
Role functioning	0.03742^a^	0.00656^a^	0.01498^a^	0.01912^a^	0.03914^a^	–	–
Emotional functioning	0.02694^a^	0.00540^a^	–	–	–	–	0.06947^a^
Cognitive functioning	–	–	–	–	–	–	–
Social functioning	–	–	–	–	0.00770	–	–
Global health status	0.03182^a^	0.01167^a^	0.01767^a^	–	0.02419^a^	0.02343^a^	–
Fatigue	–	–	–	–	–	–	–
Pain	− 0.06337^a^	–	–	–	–	− 0.07683^a^	–
Scale parameter	–	8.09789	–	–	–	–	–
FACT-G							
Intercept 1	− 11.54143	− 0.65502	− 1.86481	− 0.47810	− 5.51844	− 5.90937	− 6.77040
Intercept 2	–	–	− 0.17843	0.85700	− 3.04179	− 3.06146	− 4.14108
Intercept 3	–	–	1.19513	1.83808	− 1.60687	− 1.28905	− 2.67943
Intercept 4	–	–	3.02125	2.87152	0.22225	0.45253	− 0.88517
Age	–	− 0.01111^a^	− 0.04819^a^	− 0.04255^a^	− 0.02439	–	0.01664
Female	–	–	–	–	–	–	–
Physical well-being	0.34876^a^	0.09845^a^	0.21038^a^	0.19205^a^	0.26197^a^	0.24215^a^	0.04892^a^
Social well-being	–	–	–	–	–	–	–
Emotional well-being	0.06963	0.03355^a^	–	–	–	–	0.26968^a^
Functional well-being	0.04585^a^	0.03922^a^	0.05296^a^	0.07714^a^	0.08561^a^	0.01740	0.04819^a^
Scale parameter	–	6.05147	–	–	–	–	–

EORTC QLQ-C30, European Organization for Research and Treatment of Cancer Quality of Life Questionnaire Core 30; FACT-G, Functional Assessment of Cancer Therapy General

^a^*P* < 0.01 (not applicable to intercepts and scale parameter)

## Discussion

In the present study, we developed direct and indirect (response) algorithms that map two common cancer-specific HRQOL measures, EORTC QLQ-C30 and FACT-G, onto the EQ-5D-5L index. The recommended direct mapping algorithms for both EORTC QLQ-C30 and FACT-G are based on two-part beta regression. These direct algorithms are suitable for generating the EQ-5D-5L index based on the Japanese value set. Conversely, the recommended indirect mapping algorithms for EORTC QLQ-C30 and FACT-G are based on ordinal logistic regression and had a predictive performance that is comparable to the recommended direct mapping algorithms. These indirect mapping algorithms can generate the EQ-5D-5L index based on a value set of any country.

For EORTC QLQ-C30, several mapping algorithms onto EQ-5D-5L index were developed. The recommended direct mapping algorithm in this study has a similar predictive performance to the largest study by Lamu et al. [18], although a direct comparison is not feasible due to the different values sets used. The best model for EQ-5D-5L in Lamu et al. was derived from two-part beta regression [18], which is the same in our study. Although Lamu et al. failed to develop indirect mapping algorithms with a good predictive performance [18], we developed indirect mapping algorithms with a predictive performance that is comparable to the direct mapping algorithms using ordinal logistic regression. Our indirect mapping algorithm can be used for any value set. Our mapping algorithms have a better predictive performance than those developed by other studies, which focus on a relatively small sample of patients with a certain cancer type [19, 20].

One study has developed mapping algorithms for FACT-G onto the EQ-5D-5L index [21]. However, our recommended mapping algorithms had a better predictive performance than the previous algorithms. The recommended direct mapping algorithm for FACT-G in our study is based on two-part beta regression, which is true of EORTC QLQ-C30. Although our mapping algorithms yield a less accurate prediction of the EQ-5D-5L index than the current best mapping algorithm which additionally uses a breast cancer subscale (i.e., FACT-B) for patients with breast cancer [29], the algorithm using FACT-B cannot be used in patients with other cancers. No indirect mapping algorithm for FACT-G was available before the present study. Our indirect mapping algorithm can deal with any value set.

Although our mapping algorithms for FACT-G and EORTC QLQ-C30 have advantages over previous ones, they are not perfect. The mapping algorithms for FACT-G overestimates the mean EQ-5D-5L index in patients with an ECOG performance status of 2 and 3. This may be partly because FACT-G does not have a subscale directly relevant to pain/discomfort in EQ-5D-5L, whereas EORTC QLQ-C30 has a pain subscale. The pain subscale in EORTC QLQ-C30 has a rank correlation coefficient of > 0.7 with pain/discomfort in EQ-5D-5L and was selected as an explanatory variable in the recommended direct and indirect mapping algorithms. For indirect mapping algorithms for both EORTC QLQ-C30 and FACT-G, the proportion of full health is underestimated. This underestimation by the indirect mapping algorithms was reported for EQ-5D-3L data in other disease areas [30, 31]. A new method to solve this problem in indirect mapping would be helpful to further improve the performances of indirect mapping algorithms.

Mapping from EORTC QLQ-C30 yielded smaller overall prediction errors than mapping from FACT-G. In addition to the pain subscale mentioned above, this difference in performance could be explained by the fact that the social well-being score in FACT-G is not correlated with any item in EQ-5D-5L. It is empirically known that the social well-being subscale in FACT-G measures an aspect of HRQOL different from that measured by the social functioning subscale in EORTC QLQ-C30 [32, 33]. Besides overall prediction errors, there were two differences in the prediction performance between EORTC QLQ-C30 and FACT-G. First, FACT-G overestimated mean EQ-5D-5L index in patients with ECOG performance status 2 and 3. However, the impact of this overestimation on mapped EQ-5D-5L index may be small if mapping is applied to clinical trial data, because many patients are likely to be in relatively good condition in clinical trials. Second, the mapping algorithms for EORTC QLQ-C30 underestimated the mean EQ-5D-5L index in patients in the highest global health status group. Nevertheless, mapping algorithms for FACT-G might also underestimate the mean EQ-5D-5L index near full health, because the observed mean EQ-5D-5L index in the highest FACT-G total score subgroup was lower than in the highest global health status subgroup.

To develop the mapping algorithms, we enrolled patients with 4 major cancers (lung, stomach, colorectal, and breast) and other solid tumors. Other solid tumors included prostate cancer, ovarian cancer, cervical cancer, endometrial cancer, esophageal cancer, pancreatic cancer, renal cancer, and so on. We showed that the mean EQ-5D-5L index of the 4 major cancers could be estimated accurately by the recommended mapping algorithms, although we could not assess the accuracy in patients with cancer infrequent in our data. In addition, we did not enroll patients with hematologic cancer and patients receiving adjuvant treatment after surgical resection of cancer, whereas EORTC QLQ-C30 and FACT-G can be applied to assess the HRQOL for such patients. Although compared to previous studies, we did enroll relatively diverse patients [18–21], whether the recommended mapping algorithms can be applied to these populations needs to be explored in future research.

Although we attempted to develop mapping algorithms that used item scores rather than subscale scores [34], we did not report the detailed results because of two considerations. First, the items models for EORTC QLQ-C30 improved the predictive performance for the whole sample but did not for the cross-validated versions, suggesting an overfitting. Second, the item models for FACT-G did improve the cross-validated predictive performance too but had low face validity (i.e., many estimated regression coefficients had signs opposite to what was anticipated). Despite their improved performance, the item models for FACT-G had a lower predictive performance than the recommended subscale models for EORTC QLQ-C30. From the statistical viewpoint, subscale models stabilize estimation results by assuming the same regression coefficient for items in each subscale, since a subscale score in EORTC QLQ-C30 and FACT-G is essentially the sum of the item scores in the corresponding subscale with the same weight. Good reliability (e.g., a high Cronbach's *α*) of the two cancer-specific HRQOL measures suggests that the item scores in a subscale are highly correlated and can, thus, induce multicollinearity, which is likely to result in low face validity.

Several limitations should be taken into consideration when interpreting the results of this study. First, we used only the Japanese value set for EQ-5D-5L; thus, our direct mapping algorithms may not be suitable for cost-effectiveness analyses that use any other value set. To avoid this, we developed indirect mapping algorithms that can produce the EQ-5D-5L index based on any value set, although their performance was investigated only for the Japanese value set. This limitation is applicable to comparison of the prediction performance between EORTC QLQ-C30 and FACT-G. Second, we did not conduct external validation using external data. Third, we did not consider higher-order terms or interaction terms, which could have further improved the mapping algorithms. However, the need for higher-order and interaction terms depends on the scale of outcome. Since we used identity, log, and logit link functions while developing the mapping algorithms, some impact of higher-order and interaction terms would have been considered. Fourth, we did not apply adjusted limited dependent mixture models that show good performance for EQ-5D-3L data from patients with cancer [35, 36]. The usefulness of this method is unclear for EQ-5D-5L data that does not show multimodality and it should be investigated in future studies.

## Conclusions

Using data obtained from patients receiving drug therapy for cancer, we developed direct and indirect (response) mapping algorithms for EORTC QLQ-C30 and FACT-G onto the EQ-5D-5L index. In cost-effectiveness analyses, the developed mapping algorithms can provide the EQ-5D-5L index whose performance would be as good or better than that of previous algorithms.

## Supplementary information

**Additional file 1.** Calculation of the EQ-5D-5L index from the recommended mapping algorithms, supplementary tables, and list of hospitals participating in the QOL-MAC study.

## Data Availability

The data that support the findings of this study are available from Center for Outcomes Research and Economic Evaluation for Health, National Institute of Public Health, but restrictions apply to the availability of these data, which were used under license for the current study, and so are not publicly available.

## Abbreviations

EORTC QLQ-C30: European Organization for Research and Treatment of Cancer Quality of Life Questionnaire Core 30
FACT-G: Functional Assessment of Cancer Therapy General
RMSE: Root mean squared error
QALY: Quality-adjusted life year
HRQOL: Health-related quality of life
QOL-MAC: Quality of life mapping algorithm for cancer
ECOG: Eastern cooperative oncology group
MAE: Mean absolute error
PHRF: Public Health Research Foundation
