# Supplementary material for: Mapping EORTC QLQ-C30 and FACT-G onto EQ-5D-5L index for patients with cancer
Source: Health Qual Life Outcomes. 2020 Nov 3;18:354. doi: 10.1186/s12955-020-01611-w (PMC7641825; doi:10.1186/s12955-020-01611-w)
Supplement: Supplementary file 1 — Additional file 1. Calculation of the EQ-5D-5L index from the recommended mapping algorithms, supplementary tables, and list of hospitals participating in the QOL-MAC study. [file 12955_2020_1611_MOESM1_ESM.pdf]

Supplementary Material for "Mapping EORTC QLQ-C30 and FACT-G onto EQ-5D-5L Index for Patients with Cancer" by Y. Hagiwara, T. Shiroiwa, N. Taira, T. Kawahara, K. Konomura, S. Noto, T. Fukuda, K. Shimozuma

Table of Contents

1. Calculation of the EQ-5D-5L index from the recommended mapping algorithms
2. Supplementary Tables
3. List of hospitals participating in the QOL-MAC study

1. Calculation of the EQ-5D-5L index from the recommended mapping algorisms

1.1 Two-part beta regression for EORTC QLQ-C30

As preparation, calculate

$$\Pr(\text{Full health}) = \text{logistic}(-11.64060 + 0.03395\ \text{PF} + 0.03742\ \text{RF} + 0.02694\ \text{EF} + 0.03182\ \text{GH} - 0.06337\ \text{PA}),$$

where $\text{logistic}(x) = \{1 + \exp(-x)\}^{-1}$,

$$E(\text{transformed index}\ |\text{Not full health}) = \text{logistic}(-1.66963 + 0.15271\ \text{Female} + 0.02475\ \text{PF} + 0.00656\ \text{RF} + 0.00540\ \text{EF} + 0.01167\ \text{GH}),$$

and

$$E(\text{index}\ |\text{Not full health}) = (0.895444 + 0.025449)\ E(\text{transformed index}\ |\text{Not full health}) - 0.025449.$$

Explanation on explanatory variables (e.g., PF) is provided in the footnote of Table S1. The values 0.895444 and −0.025449 are the second largest index and the smallest index in the Japanese value set, respectively. Predicted EQ-5D-5L index as an expected value is obtained as

$$E(\text{index}) = \Pr(\text{Full health}) + \{1 - \Pr(\text{Full health})\}\ E(\text{index}\ |\text{Not full health}).$$

To simulate individual EQ-5D-5L index, generate X from the Bernoulli distribution with success probability of Pr(Full health) and Y from the beta distribution with parameters α and β, where

$$\alpha = 8.09789\ E(\text{transformed index}\ |\text{Not full health})$$

and

$$\beta = 8.09789\ \{1 - E(\text{transformed index}\ |\text{Not full health})\}.$$

Then, the simulated EQ-5D-5L index is obtained as

$$X + (1 - X)\ \{(0.895444 + 0.025449)\ Y - 0.025449\}.$$

1.2 Ordinal logistic regression for EORTC QLQ-C30

For the first item (mobility) of EQ-5D-5L, calculate

$$\Pr(\text{Mobility} \le k) = \text{logistic}\ (\gamma_{1k} + \text{LP}_1),$$

for $k = 1, 2, 3, 4$, where $\gamma_{11} = -5.91277$, $\gamma_{12} = -3.79148$, $\gamma_{13} = -1.85222$, $\gamma_{14} = 0.92106$, and

$$\text{LP}_1 = -\ 0.02469\ \text{Age} + 0.06934\ \text{PF} + 0.01498\ \text{RF} + 0.01767\ \text{GH}.$$

Pr(Mobility = k) ($k = 1, \ldots, 5$) can be calculated using these probabilities; that is,

$$\Pr(\text{Mobility} = 1) = \Pr(\text{Mobility} \le 1),$$

$$\Pr(\text{Mobility} = k) = \Pr(\text{Mobility} \le k) - \Pr(\text{Mobility} \le k - 1)$$

for $k = 2, 3, 4$, and

$$\Pr(\text{Mobility} = 5) = 1 - \Pr(\text{Mobility} \le 4).$$

This process is repeated for the other four items in EQ-5D-5L using information given in Table S1. After that, we have Pr(Mobility = k), Pr(Self-care = k), Pr(Usual activities = k), Pr(Pain/discomfort

= k), and Pr(Anxiety/depression = k) for k = 1, …, 5. Predicted EQ-5D-5L index as an expected value is obtained by weighting the disutilities in a value set by the calculated probabilities. To simulate individual EQ-5D-5L index, generate a response for each item based on categorical distributions with the calculated probabilities and then apply a value set to the simulated responses to the 5 items.

1.3 Two-part beta regression for FACT-G

As preparation, calculate

$$\Pr(\text{Full health}) = \text{logistic}(-11.54143 + 0.34876\ \text{PWB} + 0.06963\ \text{EWB} + 0.04585\ \text{FWB})$$

and

$$E(\text{transformed index}\ |\text{Not full health}) = \text{logistic}(-0.65502 - 0.01111\ \text{Age} + 0.09845\ \text{PWB} + 0.03355\ \text{EWB} + 0.03922\ \text{FWB}).$$

Explanation on variables is provided in the footnote of Table S2. E(index |Not full health) is calculated in the same manner as EORTC QLQ-C30. The next process is also the same as EORTC QLQ-C30, except for

$$\alpha = 6.05147\ E(\text{transformed index}\ |\text{Not full health})$$

and

$$\beta = 6.05147\ \{1 - E(\text{transformed index}\ |\text{Not full health})\}.$$

1.4 Ordinal logistic regression for FACT-G

The same calculation is applicable as EORTC QLQ-C30, replacing the parameter values of Table S1 with Table S2.

2. Supplementary Tables

Table S1. Parameter values used in ordinal logistic regression for EORTC QLQ-C30

Pr(Mobility $\leq k$) = logistic (γ_{1k} + LP_1)
$\gamma_{11} = -5.91277$, $\gamma_{12} = -3.79148$, $\gamma_{13} = -1.85222$, $\gamma_{14} = 0.92106$
$LP_1 = -$ 0.02469 Age + 0.06934 PF + 0.01498 RF + 0.01767 GH
Pr(Self-care $\leq k$) = logistic (γ_{2k} + LP_2)
$\gamma_{21} = -4.24535$, $\gamma_{22} = -2.56552$, $\gamma_{23} = -1.23110$, $\gamma_{24} = 0.17985$
LP_2 = 0.60145 Female + 0.06035 PF + 0.01912 RF
Pr(Usual activities $\leq k$) = logistic (γ_{3k} + LP_3)
$\gamma_{31} = -9.28712$, $\gamma_{32} = -6.21474$, $\gamma_{33} = -4.16801$, $\gamma_{34} = -1.46187$
LP_3 = 0.33291 Female + 0.04812 PF + 0.03914 RF + 0.00770 SF + 0.02419 GH
Pr(Pain/discomfort $\leq k$) = logistic (γ_{4k} + LP_4),
$\gamma_{41} = -0.82400$, $\gamma_{42} = 2.75434$, $\gamma_{43} = 5.35925$, $\gamma_{44} = 7.64850$
LP_4 = 0.02343 GH − 0.07683 PA
Pr(Anxiety/depression $\leq k$) = logistic (γ_{5k} + LP_5),
$\gamma_{51} = -6.65962$, $\gamma_{52} = -4.31221$, $\gamma_{53} = -2.81811$, $\gamma_{54} = -0.86976$
LP_5 = 0.02119 Age + 0.06947 EF
Age is on year-scale. Female takes the value of 1 if sex is female and 0 otherwise. PF, physical functioning; RF, role functioning; SF, social functioning; EF, emotional functioning; GH, global health status; PA, pain.

Tables S2. Parameter values used in ordinal logistic regression for FACT-G

Pr(Mobility $\leq k$) = logistic (γ_{1k} + LP_1)
$\gamma_{11} = -1.86481$, $\gamma_{12} = -0.17843$, $\gamma_{13} = 1.19513$, $\gamma_{14} = 3.02125$
$LP_1 = -$ 0.04819 Age + 0.21038 PWB + 0.05296 FWB
Pr(Self-care $\leq k$) = logistic (γ_{2k} + LP_2)
$\gamma_{21} = -0.47810$, $\gamma_{22} = 0.85700$, $\gamma_{23} = 1.83808$, $\gamma_{24} = 2.87152$
$LP_2 = -$ 0.04255 Age + 0.19205 PWB + 0.07714 FWB
Pr(Usual activities $\leq k$) = logistic (γ_{3k} + LP_3)
$\gamma_{31} = -5.51844$, $\gamma_{32} = -3.04179$, $\gamma_{33} = -1.60687$, $\gamma_{34} = 0.22225$
$LP_3 = -$ 0.02439 Age + 0.26197 PWB + 0.08561 FWB
Pr(Pain/discomfort $\leq k$) = logistic (γ_{4k} + LP_4),
$\gamma_{41} = -5.90937$, $\gamma_{42} = -3.06146$, $\gamma_{43} = -1.28905$, $\gamma_{44} = 0.45253$
LP_4 = 0.24215 PWB + 0.01740 FWB
Pr(Anxiety/depression $\leq k$) = logistic (γ_{5k} + LP_5),
$\gamma_{51} = -6.77040$, $\gamma_{52} = -4.14108$, $\gamma_{53} = -2.67943$, $\gamma_{54} = -0.88517$
LP_5 = 0.01664 Age + 0.04892 PWB + 0.26968 EWB + 0.04819 FWB

Age is on year-scale. Female takes the value of 1 if sex is female and 0 otherwise.

PWB, physical well-being; EWB, emotional well-being; FWB, functional well-being.

Table S3. Distributions of responses to 5 items in EQ-5D-5L

	No problem	Some problem	Moderate problem	Severe problem	Extreme problem
EORTC QLQ-C30 analysis					
Mobility	485 (53.7)	233 (25.8)	108 (12.0)	60 (6.6)	17 (1.9)
Self-care	730 (80.8)	105 (11.6)	36 (4.0)	19 (2.1)	13 (1.4)
Usual activities	426 (47.2)	295 (32.7)	104 (11.5)	58 (6.4)	20 (2.2)
Pain / discomfort	360 (39.9)	387 (42.9)	109 (12.1)	36 (4.0)	11 (1.2)
Anxiety / depression	539 (59.7)	270 (29.9)	62 (6.9)	26 (2.9)	6 (0.7)
FACT-G analysis					
Mobility	489 (53.9)	233 (25.7)	109 (12.0)	59 (6.5)	18 (2.0)
Self-care	733 (80.7)	105 (11.6)	37 (4.1)	19 (2.1)	14 (1.5)
Usual activities	424 (46.7)	301 (33.1)	101 (11.1)	60 (6.6)	22 (2.4)
Pain/discomfort	359 (39.5)	394 (43.4)	108 (11.9)	36 (4.0)	11 (1.2)
Anxiety/depression	546 (60.1)	270 (29.7)	59 (6.5)	26 (2.9)	7 (0.8)

Number (%) is reported.

Table S4. Correlation between responses to the five items in EQ-5D-5L and the subscale scores of EORTC QLQ-C30 and FACT-G

	Mobility	Self-care	Usual activities	Pain/ discomfort	Anxiety/ depression
EORTC QLQ-C30					
Physical functioning	**−0.66**	**−0.47**	**−0.65**	**−0.44**	−0.29
Role functioning	**−0.58**	**−0.46**	**−0.73**	**−0.47**	−0.32
Emotional functioning	−0.25	−0.22	−0.37	−0.36	**−0.54**
Cognitive functioning	−0.30	−0.28	−0.37	−0.31	−0.28
Social functioning	−0.37	−0.28	**−0.51**	−0.31	−0.34
Global health status	**−0.47**	−0.37	**−0.59**	**−0.47**	−0.35
Fatigue	**0.53**	0.39	**0.61**	**0.48**	0.35
Nausea and vomiting	0.27	0.17	0.29	0.29	0.25
Pain	0.36	0.34	**0.43**	**0.71**	0.29
Dyspnea	0.36	0.27	0.39	0.36	0.24
Insomnia	0.22	0.22	0.29	0.34	0.24
Appetite loss	0.38	0.29	**0.44**	0.39	0.27
Constipation	0.18	0.17	0.23	0.26	0.18
Diarrhea	0.10	0.11	0.17	0.12	0.09
Financial difficulties	0.23	0.20	0.34	0.25	0.26
FACT-G					
Physical well-being	**−0.52**	**−0.40**	**−0.64**	**−0.58**	**−0.42**
Social/family well-being	−0.04	−0.01	0.03	0.02	−0.04
Emotional well-being	−0.25	−0.20	**−0.34**	**−0.36**	**−0.58**
Functional well-being	**−0.38**	**−0.31**	**−0.48**	**−0.31**	**−0.36**

Absolute rank correlation coefficients of ≥ 0.4 for EORTC QLQ-C30 and ≥ 0.3 for FACT-G are bolded.

EORTC QLQ-C30, European Organization for Research and Treatment of Cancer Quality of Life Questionnaire Core 30; FACT-G, Functional Assessment of Cancer Therapy General.

Table S5. Variance-covariance matrix for regression coefficients of two-part beta regression for EORTC QLQ-C30

Logistic part	Intercept	PF	RF	EF	GH	PA	
Intercept	1.927692	−0.00872	−0.00354	−0.00843	−0.00045	−0.00198	
PF	−0.00872	0.000133	−0.00003	8.992E-6	−9.87E-6	7.458E-6	
RF	−0.00354	−0.00003	0.000084	−2.18E-6	−0.00001	2.584E-6	
EF	−0.00843	8.992E-6	−2.18E-6	0.000095	−0.00001	6.934E-6	
GH	−0.00045	−9.87E-6	−0.00001	−0.00001	0.000046	−1.53E-6	
PA	−0.00198	7.458E-6	2.584E-6	6.934E-6	−1.53E-6	0.000115	
Beta part	Intercept	Female	PF	RF	EF	GH	Scale
Intercept	0.019389	−0.00217	−0.00011	0.000056	−0.00016	−0.00002	-
Female	−0.00217	0.003718	1.354E-6	5.583E-6	6.527E-6	−8.55E-6	-
PF	−0.00011	1.354E-6	3.408E-6	−1.6E-6	−2.25E-7	−4.97E-8	-
RF	0.000056	5.583E-6	−1.6E-6	2.235E-6	−4.86E-7	−8.85E-7	-
EF	−0.00016	6.527E-6	−2.25E-7	−4.86E-7	3.244E-6	−9.46E-7	-
GH	−0.00002	−8.55E-6	−4.97E-8	−8.85E-7	−9.46E-7	3.04E-6	-
Scale	-	-	-	-	-	-	0.194834

PF, physical functioning; RF, role functioning; EF, emotional functioning; GH, global health status; PA, pain.

Table S6. Variance-covariance matrix for regression coefficients of ordinal logistic regression for EORTC QLQ-C30

Mobility	Intercept 1	Intercept 2	Intercept 3	Intercept 4	Age	PF	RF	GH
Intercept 1	0.346587	0.323728	0.302486	0.26437	−0.00297	−0.00187	0.00034	−0.00031
Intercept 2	0.323728	0.317973	0.295883	0.261298	−0.00303	−0.00167	0.000386	−0.00027
Intercept 3	0.302486	0.295883	0.302648	0.263204	−0.00308	−0.00143	0.000441	−0.00026
Intercept 4	0.26437	0.261298	0.263204	0.327087	−0.00311	−0.001	0.000471	−0.00022
Age	−0.00297	−0.00303	−0.00308	−0.00311	0.000045	5.096E-6	−4.16E-6	−2.22E-6
PF	−0.00187	−0.00167	−0.00143	−0.001	5.096E-6	0.000029	−9.54E-6	−4.55E-7
RF	0.00034	0.000386	0.000441	0.000471	−4.16E-6	−9.54E-6	0.000014	−5.32E-6
GH	−0.00031	−0.00027	−0.00026	−0.00022	−2.22E-6	−4.55E-7	−5.32E-6	0.000015

Self-care	Intercept 1	Intercept 2	Intercept 3	Intercept 4	Female	PF	RF
Intercept 1	0.122139	0.098178	0.080206	0.064664	−0.02614	−0.00152	−0.00005
Intercept 2	0.098178	0.099165	0.078757	0.062657	−0.02438	−0.00129	0.000017
Intercept 3	0.080206	0.078757	0.097019	0.07481	−0.02265	−0.00109	0.000054
Intercept 4	0.064664	0.062657	0.07481	0.137507	−0.02023	−0.00089	0.000067
Female	−0.02614	−0.02438	−0.02265	−0.02023	0.040994	0.000106	0.000043
PF	−0.00152	−0.00129	−0.00109	−0.00089	0.000106	0.000036	−0.00002
RF	−0.00005	0.000017	0.000054	0.000067	0.000043	−0.00002	0.000018

(Continued)

Usual activities	Intercept 1	Intercept 2	Intercept 3	Intercept 4	Female	PF	RF	SF	GH
Intercept 1	0.219022	0.170699	0.140566	0.093101	−0.01574	−0.00148	−0.00014	−0.0005	−0.00049
Intercept 2	0.170699	0.151461	0.121111	0.07994	−0.01423	−0.00127	0.000047	−0.00047	−0.00038
Intercept 3	0.140566	0.121111	0.125322	0.077513	−0.01331	−0.0011	0.000203	−0.00045	−0.00036
Intercept 4	0.093101	0.07994	0.077513	0.128403	−0.01176	−0.00074	0.000272	−0.00035	−0.00031
Female	−0.01574	-0.01423	−0.01331	−0.01176	0.02182	0.000032	0.000041	0.000022	−0.00003
PF	−0.00148	−0.00127	−0.0011	−0.00074	0.000032	0.000025	−7.92E-6	5.221E-7	−1.41E-7
RF	−0.00014	0.000047	0.000203	0.000272	0.000041	−7.92E-6	0.000018	−4.91E-6	−3.44E-6
SF	−0.0005	−0.00047	−0.00045	−0.00035	0.000022	5.221E-7	−4.91E-6	0.000013	−2.94E-6
GH	−0.00049	−0.00038	−0.00036	−0.00031	−0.00003	−1.41E-7	−3.44E-6	−2.94E-6	0.000016

Pain/discomfort	Intercept 1	Intercept 2	Intercept 3	Intercept 4	GH	PA
Intercept 1	0.06622	0.05715	0.056231	0.056218	−0.00083	−0.00041
Intercept 2	0.05715	0.080018	0.081823	0.087942	−0.00071	−0.00083
Intercept 3	0.056231	0.081823	0.138108	0.133173	−0.00064	−0.00123
Intercept 4	0.056218	0.087942	0.133173	0.252356	−0.00059	−0.00154
GH	−0.00083	−0.00071	−0.00064	−0.00059	0.000012	3.024E-6
PA	−0.00041	−0.00083	−0.00123	−0.00154	3.024E-6	0.000019

(Continued)

Anxiety/depression	Intercept 1	Intercept 2	Intercept 3	Intercept 4	Age	EF
Intercept 1	0.289513	0.262869	0.248248	0.233636	−0.00245	−0.00155
Intercept 2	0.262869	0.254951	0.238974	0.224451	−0.00238	−0.00131
Intercept 3	0.248248	0.238974	0.256207	0.236408	−0.00238	−0.00115
Intercept 4	0.233636	0.224451	0.236408	0.367939	−0.00239	−0.00096
Age	−0.00245	−0.00238	−0.00238	−0.00239	0.000038	1.678E-7
EF	−0.00155	−0.00131	−0.00115	−0.00096	1.678E-7	0.000019

PF, physical functioning; RF, role functioning; EF, emotional functioning; SF, social functioning; GH, global health status; PA, pain.

Table S7. Variance-covariance matrix for regression coefficients of two-part beta regression for FACT-G

Logistic part	Intercept	PWB	EWB	FWB
Intercept	0.884833	−0.02858	−0.00731	−0.00178
PWB	−0.02858	0.00138	−0.00023	−0.00006
EWB	−0.00731	−0.00023	0.000799	−0.00011
FWB	−0.00178	−0.00006	−0.00011	0.000265

Beta part	Intercept	Age	PWB	EWB	FWB	Scale
Intercept	0.045755	−0.00051	−0.0002	−0.00018	−0.00035	-
Age	−0.00051	8.457E-6	−2.03E-6	−2.75E-6	1.846E-6	-
PWB	−0.0002	−2.03E-6	0.000044	−0.00002	−7.98E-6	-
EWB	−0.00018	−2.75E-6	−0.00002	0.00006	−0.00001	-
FWB	−0.00035	1.846E-6	−7.98E-6	−0.00001	0.000035	-
Scale	-	-	-	-	-	0.109343

PWB, physical well-being; EWB, emotional well-being; FWB, functional well-being.

Table S8. Variance-covariance matrix for regression coefficients of ordinal logistic regression for FACT-G

Mobility	Intercept 1	Intercept 2	Intercept 3	Intercept 4	Age	PWB	FWB
Intercept 1	0.225249	0.217062	0.212301	0.208768	−0.00247	−0.00148	−0.0015
Intercept 2	0.217062	0.219365	0.213739	0.210226	−0.00256	−0.00102	−0.0014
Intercept 3	0.212301	0.213739	0.223014	0.217349	−0.00263	−0.00064	−0.00135
Intercept 4	0.208768	0.210226	0.217349	0.266206	−0.0027	−0.00028	−0.0013
Age	−0.00247	−0.00256	−0.00263	−0.0027	0.000045	−0.00003	1.437E-6
PWB	−0.00148	−0.00102	−0.00064	−0.00028	−0.00003	0.000198	−0.00004
FWB	−0.0015	−0.0014	−0.00135	−0.0013	1.437E-6	−0.00004	0.000139
**Self-care**	**Intercept 1**	**Intercept 2**	**Intercept 3**	**Intercept 4**	**Age**	**PWB**	**FWB**
Internet 1	0.386258	0.379833	0.377196	0.375758	−0.00477	−0.00114	−0.00264
Internet 2	0.379833	0.388709	0.384766	0.382722	−0.00486	−0.00067	−0.0025
Internet 3	0.377196	0.384766	0.403584	0.399667	−0.00492	−0.00041	−0.00241
Internet 4	0.375758	0.382722	0.399667	0.447825	−0.00498	−0.00022	−0.00234
Age	−0.00477	−0.00486	−0.00492	−0.00498	0.000082	−0.00004	−1.84E-6
PWB	−0.00114	−0.00067	−0.00041	−0.00022	−0.00004	0.000285	−0.00006
FWB	−0.00264	−0.0025	−0.00241	−0.00234	−1.84E-6	−0.00006	0.000266

(Continued)

Usual activities	Intercept 1	Intercept 2	Intercept 3	Intercept 4	Age	PWB	FWB
Intercept 1	0.254118	0.230739	0.219389	0.2083	−0.00226	−0.00302	−0.00199
Intercept 2	0.230739	0.225113	0.212943	0.202596	−0.00234	−0.00211	−0.00174
Intercept 3	0.219389	0.212943	0.218299	0.205042	−0.00237	−0.00159	−0.00165
Intercept 4	0.2083	0.202596	0.205042	0.242338	−0.0024	−0.00107	−0.00154
Age	−0.00226	−0.00234	−0.00237	−0.0024	0.00004	−0.00002	3.055E-6
PWB	−0.00302	−0.00211	−0.00159	−0.00107	−0.00002	0.00023	−0.00003
FWB	−0.00199	−0.00174	−0.00165	−0.00154	3.055E-6	−0.00003	0.000144

Pain/discomfort	Intercept 1	Intercept 2	Intercept 3	Intercept 4	PWB	FWB
Intercept 1	0.107225	0.079981	0.067202	0.060102	−0.00373	−0.00113
Intercept 2	0.079981	0.071563	0.058211	0.051603	−0.00273	−0.00107
Intercept 3	0.067202	0.058211	0.070154	0.060128	−0.00218	−0.00106
Intercept 4	0.060102	0.051603	0.060128	0.131946	−0.00186	−0.00106
PWB	−0.00373	−0.00273	−0.00218	−0.00186	0.000216	−0.00006
FWB	−0.00113	−0.00107	−0.00106	−0.00106	−0.00006	0.000132

(Continued)

Anxiety/depression	Intercept 1	Intercept 2	Intercept 3	Intercept 4	Age	PWB	EWB	FWB
Intercept 1	0.314163	0.280924	0.268241	0.258677	−0.00283	−0.00173	−0.00331	−0.00234
Intercept 2	0.280924	0.270928	0.256536	0.246795	−0.00277	−0.0015	−0.00218	−0.0022
Intercept 3	0.268241	0.256536	0.273555	0.259319	−0.00274	−0.00139	−0.00175	−0.00212
Intercept 4	0.258677	0.246795	0.259319	0.375502	−0.00272	−0.0013	−0.00143	−0.00203
Age	−0.00283	−0.00277	−0.00274	−0.00272	0.000044	−2.14E-6	−0.00001	0.000012
PWB	−0.00173	−0.0015	−0.00139	−0.0013	−2.14E-6	0.000221	−0.00011	−0.00005
EWB	−0.00331	−0.00218	−0.00175	−0.00143	−0.00001	−0.00011	0.000419	−0.00002
FWB	−0.00234	−0.0022	−0.00212	−0.00203	0.000012	−0.00005	−0.00002	0.000184

PWB, physical well-being; EWB, emotional well-being; FWB, functional well-being.

3. List of hospitals participating in the QOL-MAC study

Name of hospitals and a supporting entity	Number of enrolled patients
Tokyo Metropolitan Cancer and Infectious Diseases Center Komagome Hospital	127
Gunma Prefectural Cancer Center	205
Shonan Kamakura General Hospital	23
NTT Medical Center	75
Edogawa Hospital	114
Japanese Redcross Medical Center	73
Tokai University School of Medicine	10
Aichi Cancer Center Hospital	168
Showa University Northern Yokohama Hospital	29
Okayama University Hospital	30
Cancer Institute Hospital, Japanese Foundation for Cancer Research	99
Tsuboi cancer center Hospital	27
Kosei Chuo General Hospital	21
Toranomon Hospital	30
Qol Co., Ltd.	(Support for recruiting patients)
